# Nanobiotechnology can boost crop production and quality: first evidence from increased plant biomass, fruit yield and phytomedicine content in bitter melon (*Momordica charantia*)

**DOI:** 10.1186/1472-6750-13-37

**Published:** 2013-04-26

**Authors:** Chittaranjan Kole, Phullara Kole, K Manoj Randunu, Poonam Choudhary, Ramakrishna Podila, Pu Chun Ke, Apparao M Rao, Richard K Marcus

**Affiliations:** 1 Department of Genetics and Biochemistry and Institute of Nutraceutical Research, Clemson University, Clemson, SC, USA; 2 Department of Chemistry, Clemson University, Clemson, SC, USA; 3 Department of Physics and Astronomy, Clemson University, Clemson, SC, USA; 4 Present address: Vice-Chancellor, Bidhan Chandra Krishi (Agricultural) Viswavidyalaya (University), Mohanpur, West Bengal, India

**Keywords:** Nanoparticles, Fullerol, Bitter melon, Seed treatment, Uptake, Accumulation, Fruit yield, Plant biomass, Phytomedicine content, Water content

## Abstract

**Background:**

Recent research on nanoparticles in a number of crops has evidenced for enhanced germination and seedling growth, physiological activities including photosynthetic activity and nitrogen metabolism, mRNA expression and protein level, and also positive changes in gene expression indicating their potential use in crop improvement. We used a medicinally rich vegetable crop, bitter melon, as a model to evaluate the effects of seed treatment with a carbon-based nanoparticle, fullerol [C_60_(OH)_20_], on yield of plant biomass and fruit characters, and phytomedicine contents in fruits.

**Results:**

We confirmed the uptake, translocation and accumulation of fullerol through bright field imaging and Fourier transform infra-red spectroscopy. We observed varied effects of seed treatment at five concentrations, including non-consequential and positive, on plant biomass yield, fruit yield and its component characters, and content of five phytomedicines in fruits. Fullerol-treatment resulted in increases up to 54% in biomass yield and 24% in water content. Increases of up to 20% in fruit length, 59% in fruit number, and 70% in fruit weight led to an improvement up to 128% in fruit yield. Contents of two anticancer phytomedicines, cucurbitacin-B and lycopene, were enhanced up to 74% and 82%, respectively, and contents of two antidiabetic phytomedicines, charantin and insulin, were augmented up to 20% and 91%, respectively. Non-significant correlation *inter se* plant biomass, fruit yield, phytomedicine content and water content evidenced for separate genetic control and biosynthetic pathways for production of plant biomass, fruits, and phytomedicines in fruits, and also no impact of increased water uptake.

**Conclusions:**

While our results indicated possibility of improving crop yield and quality by using proper concentrations of fullerol, extreme caution needs to be exercised given emerging knowledge about accumulation and toxicity of nanoparticles in bodily tissues.

## Background

During the last decade, an array of exploratory experiments has been conducted to gauge the potential impact of nanotechnology on crop improvement. Two comprehensive reviews have presented evaluation of a variety of nanomaterials (NMs), mostly metal-based (MBNMs) and carbon-based (CBNMs), for their absorption, translocation, accumulation, and importantly, effects on growth and development in an array of crop plants [1,2]. Some of these studies have documented non-consequential or negative effects on plant growth and development upon NM exposure, whereas others report positive results. The positive morphological effects included enhanced germination percentage and rate; length of root and shoot, and their ratio; and vegetative biomass of seedlings in many crop plants including corn, wheat, ryegrass, alfalfa, soybean, rape, tomato, radish, lettuce, spinach, onion, pumpkin and cucumber. Enhancement of many physiological parameters related to plant growth and development were also reported that include enhanced photosynthetic activity and nitrogen metabolism by MBNMs in a few crops including soybean [3], spinach [4-8], and peanut [9] and by multiwalled carbon nanotubes (MWCNTs) in tomato [10]. Only recently, the genetic implications of such nanoparticle-induced positive changes have been validated through investigations on enhanced mRNA expression and protein level in spinach [6] by nano-TiO_2_, generational transmission of fullerol through seeds in rice [11], and changes in gene expression at plant and cellular level in tomato and tobacco [12,13] by MWCNTs. Despite such promise towards enhanced plant growth and development, there is only one report on the improvement of agronomic traits that documented increased leaf and pod dry weight and grain yield of soybean by exposure to nano-iron oxide [14].

In the meantime, concerns have been raised about potential adverse effects of nanoparticles on biological systems and the environment [15,16]. However, owing to their mutual interaction, CBNMs aggregate readily and are not considered potential contaminants in liquid phase [17]. Besides, MWCNTs could be reportedly water-stabilized by Suwannee River, Georgia through vigorous agitation [18]. Pristine fullerenes and MWCNTs could also be stabilized by dissolved organic matter extracted from the Sahan River, Ukraine, or by dissolved humic and tannic acids [19-21]. A fullerene derivative C_60_(OH)_20_, or “fullerol”, is readily water-soluble and known for its antioxidative effects on mammalian cells; but damages onion cells [22,23]. Furthermore, the antioxidant, antiviral, and anticancerous activities of fullerenes and their derivatives were reported [24-26], which were attributed to suppressed accumulation of superoxide- and hydroxyl radical-initiated lipid peroxidation as well as the initiation of free radical-scavenging activities of the nanoparticles. Collectively, these studies suggest fullerol, upon environmental release, could result in favorable effects on crop yield and quality; the topic addressed in the current study.

The effects of fullerol on agroeconomic traits in bitter melon (*Momordica charantia*) are presented herein. We used this specialty cucurbit crop, because it is cultivated in many tropical countries as a source of both vegetable and medicine. It contains over 60 phytomedicines [27] (listed at http://http//www.rain-tree.com/bitmelon.htm) having medicinal properties and actions against nearly 30 human diseases, including cancer, diabetes and AIDS [27-29]. Hence, demonstration of any increase of its fruit yield and/or phytomedicine content through nanobiotechnological intervention could be useful to follow as a model for other crops. Production of higher plant biomass as a feedstock for bioenergy production has recently emerged as an important target in agriculture [30]. Increase in biomass yield could facilitate the use of plant residues, such as stems and leaves, even after harvesting the consumable economic products in grain and fruit crops. We report here on the improvement in biomass yield, and fruit yield along with its component characters, coupled with enhanced content of four anticancer and antidiabetic phytomedicines realized through seed treatment with fullerol. Also included is verification of the role of plant water content on the improvement in biomass yield, fruit yield, and phytomedicine content in fruits.

## Results

### Fullerol suspension characterization

**Figure 1:**
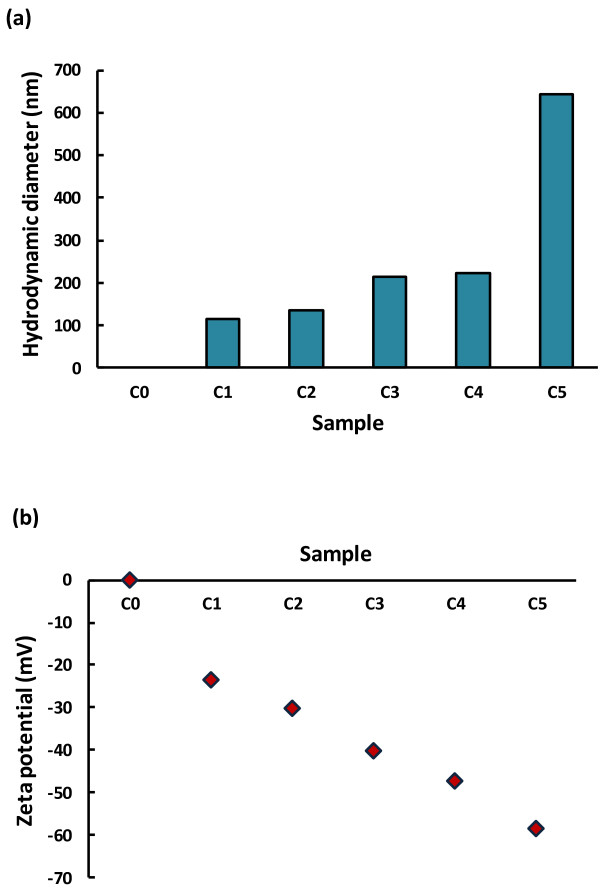
**Characterization of fullerol suspension.** (**a**) Hydrodynamic sizes of fullerols of 0.943, 4.72, 9.43, 10.88, and 47.2 nM (C1-C5). C0 denotes the control. (**b**) Zeta potential of fullerols of 0.943, 4.72, 9.43, 10.88, and 47.2 nM (C1-C5). C0 denotes the control.

Figure 1a reflects an increase in hydrodynamic size with increasing fullerol concentration (0.943, 4.72, 9.43, 10.88, and 47.2 nM), resulting from the aggregation of fullerols through hydrogen bonding. In a separate procedure, fullerol suspension of 9.43 μM (pH = 6.5, in Milli-Q water) was bath-sonicated for 15 min (Branson 1510) and filtration was applied to the suspension with Anotop filters (0.1 μm, Whatman). The hydrodynamic diameters of the fullerols were then determined to be 1.5 ± 0.2 nm and 5.0 ± 0.7 nm. These much smaller-sized nanoparticles, whose scattering was skewed in the initial DLS measurement without filtration, are believed to have contributed appreciably to the uptake of nanoparticles in the plants.

The zeta potentials of the fullerol suspensions remained negative for all concentrations, indicating good solubility of the nanoparticles (Figure 1b). Such negative charge of C_60_(OH)_20_ is attributed to the bond stretching or deprotonation of the hydroxyl groups of the nanoparticle in the polar solvent of water.

**Figure 2:**
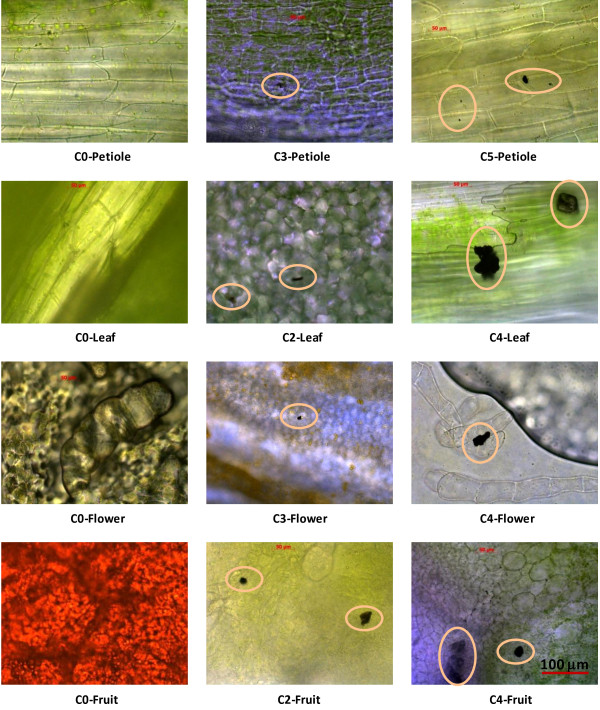
**Biodistribution of fullerols in plant organs including petioles, leaves, flowers, and fruits.** The circles highlight black aggregates which were later confirmed by FTIR as fullerols.

The biodistribution of the fullerols was examined using bright field imaging, where dark spots (Figure 2) were observed under the microscope and were later confirmed to be fullerol clusters using Fourier transform infra-red (FTIR) spectroscopy.

### FTIR Spectroscopic measurements

**Figure 3:**
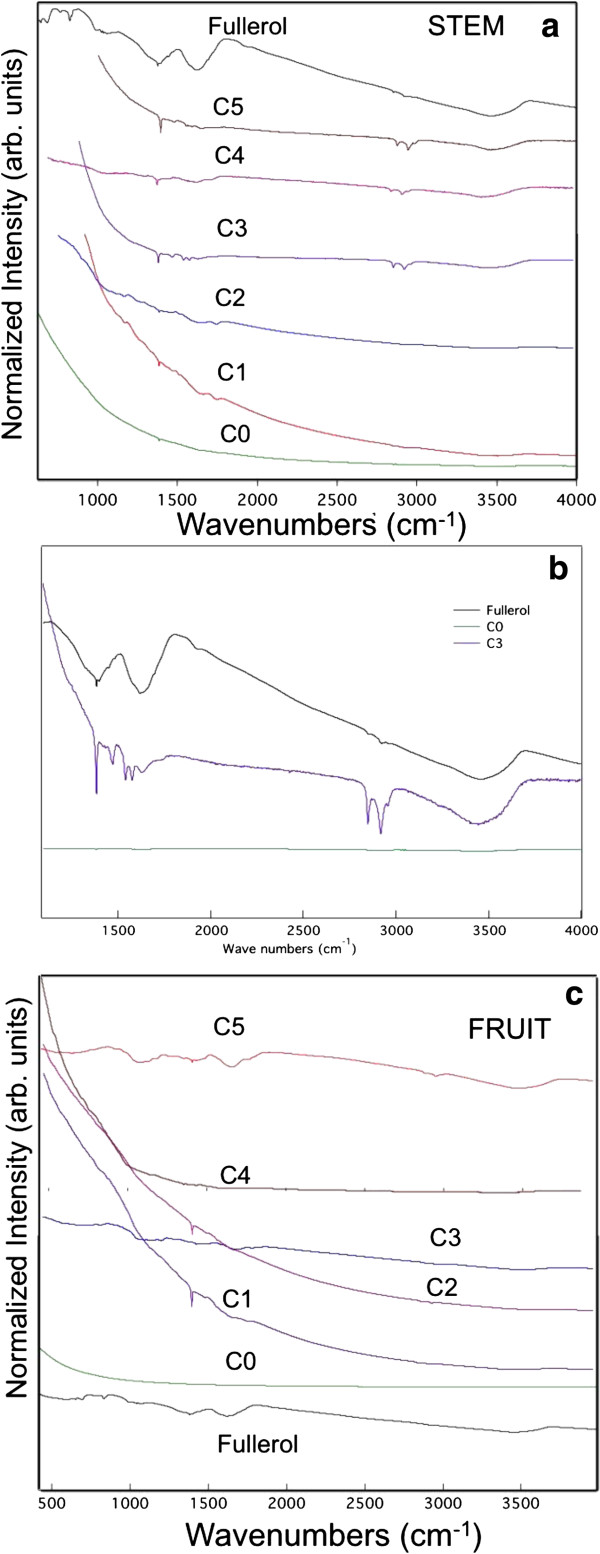
**FTIR spectroscopy of fullerols in plant organs.** (**a**) FTIR data for fullerols, C0-C5 stem samples. C1-C5 samples exhibit clear fullerol signatures. All the spectra were offset for clarity. (**b**) A scaled and expanded view of C3 sample showing the fullerol peaks ~1580-1640 cm^-1^ region. (**c**) FTIR data for fullerols, C0-C5 fruit samples. C1-C5 samples exhibit clear fullerol signatures. All the spectra were offset for clarity. Sample C5 shows very distinct features similar to fullerols due to preliminary incubation of seeds in highest fullerol concentration.

As shown in Figure 3a, pristine fullerols exhibited several distinct infra-red (IR) absorption features. It is worthwhile to note that some of these features (see Additional file 1: Table S1) may be observed commonly in any biomass and cannot be used for conclusive identification of fullerols. Specifically, some of the plant parts, such as leaf and roots, exhibited strong background in FTIR spectra due to their natural organic content, overwhelming the fullerol signature. However, the peaks present at ~1585 and 1640 cm^-1^ are unique to fullerols, arising from tangential stretching of carbon atoms and C-OH stretching, respectively [31-33]. The presence of these unique IR features in our stem and fruit spectra are taken as confirmation of the presence of fullerols in the samples. Figure 3 shows the typical IR features for pristine fullerols in C0-C5 stem and fruit samples.

### Changes in fruit yield and component characters

**Figure 4:**
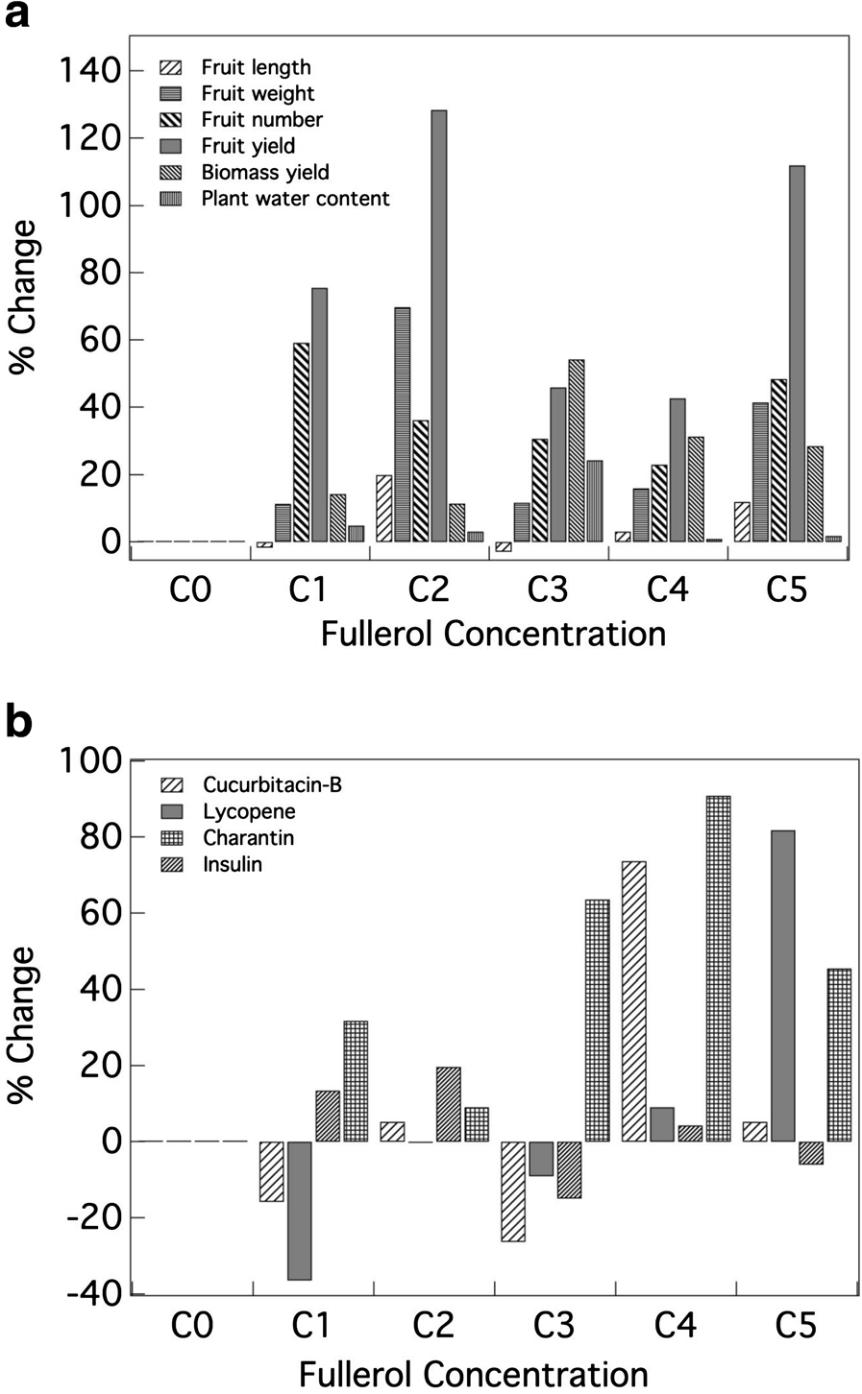
**Changes in the variables due to seed treatment with fullerol at five concentrations (C1 to C5).** (**a**) Changes (in%) in six plant characters over the control (C0). (**b**) Changes (in%) in content of four phytomedicines in fruits over the control (C0).

All of the five fruit-related characters studied, except fruit diameter, exhibited significant variation (*P*-values ranging from <0.001 to 0.0405) among the six fullerol concentrations (Table 1). However, analysis of variance (ANOVA) did not exhibit any variation among the three replications. Seed treatment with fullerol resulted in significant increase in fruit yield for all the five concentrations (Table 2, Figure 4a). C2 at par with C5 produced the highest yield with an increase of 128.45% and 112.05%, respectively over the control (C0). These were followed by C1, which in turn outyielded C3 and C4 that were at par and surpassed the control. Similarly, all the five concentrations led to significant increase in fruit weight. C2 with an increase of 69.8% outweighed C5 (41.44% increase) but both superseded C1, C3 and C4 that were at par. For fruit length, only C2 showed significant superiority over the control with an increase of 20% and was on par with C5 that also significantly superseded the control (11.96%) although was on par with C4. Other three concentrations, C1, C3 and C4 were on par *inter se* and with the control. Only three concentrations, viz., C1, C5 and C4 significantly outnumbered the control with increases of 59.23%, 48.46% and 36.15%, respectively but were statistically on par with C3 (30.77%). However, C5, C2, C3 and C4 were statistically on par, and C3 and C4 were on par with the control.

**Table 1:** Statistical data on phenotypic variation in seven plant characters and content of five phytomedicines

**Variables**	**Range**	**Grand mean**	***F*-Value**^**a**^	***P*-value**^**b**^
Fruit length (cm)	4.51-5.98	5.097	11.938**	0.0006
Fruit diameter (cm)	2.64-3.12	2.890	2.449	0.1069
Fruit weight (g)	7.50-13.78	9.866	65.197**	<0.0001
Fruit number	12.00-23.00	17.283	3.594*	0.0404
Fruit yield (g)	91.84-244.49	171.855	54.743**	<0.0001
Biomass yield (Kg)	0.03-0.06	0.043	28.753**	<0.0001
Plant water content (Kg)	0.21-0.29	0.239	9.380**	0.0016
Cucurbitacin-B content (mg/g)	0.10-0.37	0.203	7.148**	0.0043
Lycopene content (mg/g)	0.01-0.02	0.012	4.908*	0.0158
β-Carotene content (mg/g)	0.90-1.65	1.293	1.068	0.4326
Charantin content (mg/g)	5.01-8.29	6.513	3.647*	0.0388
Insulin content (mg/g)	0.19-0.52	0.308	3.404*	0.0469

^a^calculated with *n*_*1*_ = 5 for treatment *d.f.* and *n*_*2*_ = 10 for error *d.f.*^b^converted for one-tail value from *F*-value * and ** denotes significant at 1% and 5% level, respectively.

**Table 2:** Statistical comparison of the effect six fullerol concentrations on six plant characters and content of four phytomedicines based on mean values of the concentrations

**Variables**	**C0**	**C1**	**C2**	**C3**	**C4**	**C5**	**SEm**	**CD**^**a**^
Fruit length (cm)	4.85	4.77	5.82	4.71	5.00	5.43	0.128	0.402
Fruit weight (g)	7.89	8.79	13.40	8.81	9.15	11.16	0.253	0.797
Fruit number	13.00	20.70	17.70	17.00	16.00	19.30	1.413	4.452
Fruit yield (g)	102.63	180.22	234.46	149.78	146.41	217.63	6.624	20.873
Biomass yield (Kg)	0.035	0.040	0.039	0.054	0.046	0.045	0.001	0.004
Plant water content (Kg)	0.226	0.237	0.233	0.281	0.228	0.230	0.007	0.021
Cucurbitacin-B content (mg/g)	0.19	0.16	0.20	0.14	0.33	0.20	0.025	0.080
Lycopene content (mg/g)	0.011	0.007	0.011	0.010	0.012	0.020	0.002	0.006
Charantin content (mg/g)	6.34	7.19	7.59	5.39	6.61	5.96	0.421	1.327
Insulin content (mg/g)	0.22	0.29	0.24	0.36	0.42	0.32	0.041	0.129

C0 denotes control, C1 to C5 denote five fullerol concentrations. ^a^calculated from multiplying SEm value by√2 and *t*-value of 2.2281 at 5% level of significance.

### Changes in biomass yield

Biomass exhibited significant variation (*P*-value <0.001) among the six concentrations (Table 1). The highest increase in biomass yield was at C3 that led to an increase of 54.29% over the control (Table 2, Figure 4a). It was followed by C4 and C5, which were at par and resulted in increase of 31.43% and 28.57% over the control, respectively. The control produced the least biomass yield but was at par with C2.

### Changes in plant water content

Plant water content exhibited significant variation (*P*-value 0.0016) among the six concentrations (Table 1). Similar to biomass yield, C3 again superseded all the four concentrations and the control with regard to water content (Table 2, Figure 4a). It led to an increase of 24.34% over the control. All the remaining four concentrations were at par with the control.

### Changes in phytomedicine content

The content of the five phytomedicines, except β-carotene, exhibited significant (*P*-vales ranging from 0.0043 to 0.0469) variation (Table 1). Fullerol treatment resulted in the highest cucurbitacin-B content at C4 showing an increase of 73.68% over the control (Table 2, Figure 4b). The remaining four concentrations were on par *inter se* and with the control. Lycopene content was the highest at C5 exhibiting an increase of 81.82% over the control, while the remaining concentrations were on par *inter se* and the control. The highest content of charantin was obtained at C2 with an increase of 19.72% but C2 was at par with C1, C4 and C0, which were at par with C5 and C3. Insulin content was highest at C4 with an increase of 90.91% but was on par with C3 (63.64%) and C5 (45.45%), which were on par with C1 and C2. C5, C1 and C2 were at par *inter se* and with the control.

### Correlation among fruit traits, plant biomass, yield and phytomedicine contents

Correlation analysis *inter se* biomass yield, fruit traits, phytomedicine contents and plant water content (Additional file 2: Table S2) revealed significant association only between fruit length and fruit weight (*P* = 0.0021) and between fruit weight and fruit yield (*P* = 0.0148). There was no correlation between fruit traits, biomass yield, phytomedicine content and plant water content. However, statistically non-significant but considerably high correlation was observed for fruit yield with fruit length (*P* = 0.0565) and fruit number (*P* = 0.0967), and biomass yield with insulin (*P* = 0.0649) and plant water content (*P* = 0.0655).

## Discussion

Our bright field imaging and FTIR spectroscopy analysis clearly indicated the absorption and translocation of fullerols in the plant organs (roots, stems, petioles, leaves, flowers, and fruits), and their generational transmission, consistent with an earlier study on the uptake of fullerene C_70_ (suspended in natural organic matter) in rice [11]. Most of the stem and fruit samples (excluding C0 and C1) exhibited distinct FTIR features common to fullerols across the 1500–1700 cm^-1^ spectral region (see Figure 3b), suggesting the presence of fullerols in the samples. Importantly, fullerol-like IR features were absent in sample C0, obviously reflecting the absence of the nanomaterial. As seen in Figure 3c, only the fruits from C3 and C5 samples exhibited intense FTIR signal for fullerols. This result is expected since the C5 seeds were treated at the highest fullerol concentration. The major mechanism for the uptake of fullerol in our study is believed to be transpiration resulting from the water evaporation from the shoot organs, concentration gradient of the nanoparticles within the plant continuum, as well as hydrophobic interaction between the nanoparticles and the waxy layers between the plant cells (see Figure 1, panels for C3- and C5-petiole and C2-leaf).

The results revealed that seed treatment with fullerol at different concentrations led to varying effects on biomass, fruit characters and phytomedicine content. The extent of these effects also varied significantly. Among the five different fullerol concentrations, C2 promoted the highest fruit yield and its component characters, whereas C3 produced the highest biomass yield. C2, C4, and C5 led to increased contents of charantin, cucurbitacin-B, insulin, and lycopene, respectively. In all cases, the remaining concentrations either superseded or were on par with the control. Moreover, the same individual concentrations produced effects of different directions and degrees on different variables. Therefore, selection of proper concentration of nanoparticle is important for realizing higher benefits for a target agroeconomic trait. Two exhaustive lists of positive or non-consequential effects and negative effects of nanoparticles on different food crops presented in a recent review [2] substantiate our findings. It exemplified that the nanoparticles which were of same sizes and treated by similar methods could produce three types of effects on the same seedling trait in the same crop species. Besides, the effects were different in different seedling parameters such as germination, root length, shoot length and their ratios. While fullerols show no effect on mammalian cell viability [22,23], at 70 mg/l, they induced 5% cell damage in onion after 9 h of incubation as a result of their accumulation between the rigid cell walls and the fluidic plasma membranes. In contrast, the more hydrophobic fullerene C_70_ nanoparticles were largely retained by the cell-walls and elicited no toxicity [23]. Other previous works deliberated in two recent reviews [1,2] also report similar variability in effects of nanopartciles on plant growth and development. It is evident then that independent genetic regulation exists for the biosynetheic and physiological pathways for production of biomass, fruits, and phytomedicines in fruits.

Exploratory research on the positive impacts of nanoparticles on plant growth and development and the underlying physiological and genetic factors have been conducted mostly at seedling stages [1,2]. To the best of our knowledge, improvement of any agronomic yield was reported only in one instance in soybean [14], wherein increased leaf and pod dry weight resulting in a 48% increase in grain yield by nano-iron oxide treatment was reported. However, this report does not decipher the causal factors for such increases. We also observed strikingly high enhancement in biomass yield, fruit yield, and phytomedicine content by fullerol treatment at different concentrations. However, with the available data, it is not possible to precisely decipher the causal physiological and genetic factors underlying such genetic improvements. However, a previous study in tomato [10] indicated that seeds exposed to MWCNTs had higher level of moisture as compared to the untreated seeds. The authors hypothesized that their observed enhanced germination parameters, including germination rate, length of stem and fresh vegetative biomass, were based on the role of the carbon nanotubes in the process of water uptake inside the seed embryo. Therefore, we verified the plausible association of plant water content with the effects on biomass yield, fruit yield and its component characters, and phytomedicine content. However, we observed no significant correlation of plant water content with the agro-economic traits including biomass yield, fruit yield and phytomedicine contents in fruits. On the other hand, we observed that plant water content had a non-significant, but highly positive, association with biomass yield.

Reviews on previous research provide evidence for enhancement of various physiological factors related to photosynthesis and nitrogen metabolism [1,2,34]. Earlier, nitrate reductase activity was reported to increase the absorption and utilization of water/fertilizer and enhanced antioxidant system using a mixture of nano-SiO_2_ and TiO_2_ in soybean [3]. These might be the physiological mechanisms underlying the increased germination and shoot growth in their experiment. Exposure to nano-TiO_2_ in spinach resulted in increased chlorophyll formation, ribulosebiphosphate carboxylase/oxygenase activity and acceleration of the rate of evolution of oxygen in the chloroplasts that could have promoted photosynthesis leading to increased germination, germination and vigor indices, and ultimately plant dry weight [4,5]. From the follow-up studies, the authors reported enhanced activity of rubisco activase, rubisco carboxylation, rate of photosynthetic carbon reaction and chlorophyll content that could have resulted in increased plant dry weight [6,7]. From a later study in spinach, nano-TiO_2_ treatment was found to improve light absorbance, transformation from light energy to electron energy and chemical energy, and promoted carbon dioxide assimilation [8]. Magnetic nanopartciles coated with tetramethylammonium hydroxide also led to an increase in chlorophyll-a level in maize [35]. Recently, use of iron-oxide was claimed as facilitators for iron and photosynthate transfer to the leaves of peanut [9]. Use of iron-oxide in pumpkin was also observed to increase root elongation that was attributed to the Fe-dissolution [36].

There are few, but highly suggestive, reports on genetic implication for changes in plant growth and development due to nanoparticle-treatment. Germinating maize seeds in presence of magnetic fluid followed by exposure to electromagnetic field was observed to cause a pronounced increase in nucleic acid level due to the regeneration reactions of plant metabolism processes [37]. Nano-TiO_2_ treatment led to a highly enhanced mRNA expressions and protein level in spinach [6]. Expression of several water channel genes including important prolactin-induced protein (PIP) genes was characterized during rice seed germination [38]. Recently, it has been deciphered that MWCNTs induce novel changes in gene expression in tomato leaves and roots, particularly up-regulation of the stress-related genes including those induced by pathogens and the water channel *LeAqp2* gene employing microarray analysis of transcripts [12]. In a later extensive study in tobacco, these authors have detected a correlation between activation of growth of cells exposed to MWCNTs and up-regulation of genes underlying cell division and cell wall formation, and water transport [13]. They also observed expression of tobacco aquaporin gene (*NtPIP1*) along with production of the *NtPIP1* protein, significantly increased in cells exposed to MWCNTs compared to the control cells. They also detected up-regulation of expression of marker genes for cell division (*CycB*) and cell wall extension (*NtLRX1*) in the exposed cells.

## Conclusions

In the present study, we demonstrated the accumulation of fullerol in tissues and cells of root, stem, petiole, leaf, flower and fruit at particular concentrations as the causal factor of increase in biomass yield, fruit yield and phytomedicine content in fruits. These findings could pave the way for further physiological, genomics, transcriptomics and metabolomics studies underlying genetic causes for promotion of such agroeconomic characters. The concepts and strategies of nanobiotechnology of the present study could also be employed for validation and exploitation in other crops for augmentation of yield and amelioration of quality related to food, feed, fiber, fuel, aesthetics, and health, etc.

## Methods

### Fullerol suspension preparation and characterization

Fullerol, C_60_(OH)_20_, nanoparticles (BuckyUSA) were dissolved in Milli-Q water (pH 6.5) to prepare five stock concentrations (0.943, 4.72, 9.43, 10.88, and 47.2 nM), referred to hereinafter as C1, C2, C3, C4, and C5, respectively. Only Milli-Q water, without any fullerol, served as the control (C0). The hydrodynamic diameters of fullerol in the suspensions were determined at room temperature using a dynamic light scattering (DLS) device (Nanosizer S90, Malvern Instruments). The zeta potentials of the nanoparticle suspensions were measured using a Zetasizer (Nano-ZS, Malven Instruments).

### Seed treatment and growing of plants

Thirty-six uniform and healthy seeds of a bitter melon variety, CBM12, developed by C. Kole and his coworkers at Clemson University (USSN: 13/179,952) were used in this study. Five lots of seeds, with six seeds in each, were treated in fullerol solutions at the above-mentioned concentrations for 48 hours. One lot of six seeds was kept in Milli-Q water to serve as the control. Six germinated seeds from each of these six lots were planted one each in a 3-gallon pot (10.5″ diameter, 9.5″ height) filled with a 3B potting mix (Fafard) and two such pots were placed in each of three benches serving as three replications in a greenhouse. The plants were grown following recommended [39] cultural practices under uniform conditions of temperature (80/65 °F at day/night), relative humidity (70%) and photoperiod (16/8 h light/dark). Pots were watered once in a day. A Peter Excel (Everris) fertilizer solution of 15:15:15 of N, P and K, respectively (Scotts Corp.) was applied in the pots once in a week. Each plant was provided about 40″ × 24″ spacing on the greenhouse benches (Ludy Greenhouse MFG Corp.). on.

### Bright-field imaging of fullerol uptake by plants

Sections from the roots, stems, leaves, petioles, flowers, and fruits of the plants were taken; for the non-stem or -root portions of the plants, the parts closest to the plant roots were selected. The samples were then washed with de-ionized water and cut into thin cross-sections for imaging with a 40× objective of bright-field microscopy (Imager A1, Zeiss).

### FTIR spectroscopic measurements

FTIR spectroscopic measurements were performed using a Bruker-IFS v66 spectrophotometer in the transmission mode. For these measurements, 2 mg of each sample was mixed with 98 mg of KBr and pressed into a pellet.

### Recording of data on plant parameters

All well-matured, green unripe fruits of each of the two plants in each replication of each concentration were harvested over a period of 85 days, with a few fruits in each plant allowed to ripen for later collection. Average length (cm), average maximum diameter (cm), average weight (g), total number and total yield of unripe fruits were recorded for each plant. Each entire plant, except roots, was weighed after harvesting of fruit to obtain fresh plant weight (kg). These plants were collected in paper bags and kept in an oven at 100°C for 10 days to obtain plant biomass yield (kg). The water content (kg) of each plant was deduced by subtracting plant biomass yield from the fresh plant weight. All metric data recorded on each plant for each of the seven plant parameters were finally averaged to obtain per-plant data for each replication under each concentration.

### Extraction and quantification of phytomedicines

The unripe and ripe fruits from two plants for at each fullerol concentration were chopped, lyophilized (Labconco Freeze-Zone 2.5) and ground to powder in liquid nitrogen. The bioactives were extracted from the powders following a pressurized liquid extraction method [40]. Briefly, 1.0 g of powder was used for extraction in 100% methanol at solvent flow rates of 2–6 ml/min at 100°C and 1000 psi, with 9 ml of extract collected. Extracts were freeze-dried (Labconco Freeze-Zone 2.5) and re-suspended in 1.0 ml of 1:1 chloroform:methanol. The suspensions were centrifuged at 6000 rpm for 5 min and the supernatant filtered (0.45 μm, VWR). Identification and quantification of the phytomedicines was performed on high performance liquid chromatography (HPLC) system (Waters 600S) fitted with 616 HPLC pump and 996 photodiode array detector (Milford, MA, USA), employing a Zorbax Eclipse XDB-phenyl column (4.6 x 250 mm, 5 μm; Agilent Technologies). A gradient program held at A = 95% for 5 min, 95%-5% A in 20 min (4.5%/min) and 5% A for 25 min (A = 0.1% TFA in water and B = 100% methanol) at a mobile phase flow rate of 1 ml/min was employed in all cases. Data was collected by the Empower 2 Chromatography Manager and further processed and managed in Microsoft Excel. Phytomedicine standards including cucurbitacin-B, lycopene, β-carotene charantin (Chromadex) and bovine insulin (Sigma) were used to identify the peaks and construct the calibration curves for each standard. The peak area was measured at the respective wavelength of 235, 250, 450 205 and 280 nm, respectively for each phytomedicine and was converted to mg per gram of powder used for extraction using the calibration curves. The content of the phytomedicines did not differ significantly between the fresh and ripe fruits and therefore data on only the ripe fruits were analyzed and presented.

### Statistical analysis

Analysis of variance (ANOVA) for each of these seven plant parameters and five phytomedicine contents was performed following routine statistical analysis for a randomized complete block design. Significance of variation among treatments and replications was tested at 5% and 1% level of significance. Critical difference (CD) values were computed for each plant parameter for comparison between concentrations by multiplying standard error of mean by√2 and *t*-value of 2.2281 at 5% level of significance. The mean values over three replications of biomass yield; length, weight, number and yield of fruits; contents of cucurbitacin-B, lycopene, charantin and plant insulin; and water content that showed significant variation were used to compute their *inter se* correlation. Pearson correlation coefficients between these variables were computed following routine statistical procedure and tested for significance at 5% and 1% level. Changes in the plant parameters and phytomedicine content upon nanoparticle treatment at each of the five concentrations (over the control) were expressed as percentages.

## Abbreviations

AIDS: Acquired immunodeficiency syndrome; ANOVA: Analysis of variance; ANOVA: Analysis of variance; CBNMs: Carbob-based nanomaterials; CD: Critical difference; DLS: Dynamic light scattering; FTIR: Fourier transform infra-red; HPLC: High pressure liquid chromatography; IR: Infra-red; MBNMs: Metal-based nanomaterials; MWCNTs: Multiwalled carbon nanotubes; NMs: Nanomaterials; PIP: Prolactin-induced protein.

## Competing interests

The authors declare that they have no competing interests.

## Authors’ contributions

PC and PCK contributed to the bright-field imaging, zeta potential and dynamic light scattering measurements; RP and AMR conducted the FTIR spectroscopy; CK and PK evaluated the effects on biomass, fruit traits and content of phytomedicines; and KMR and RKM assisted in the estimation of the content of phytomedicines. CK prepared the manuscript with assistance from PCK, RP and AMR on biophysical aspects. CK conceived and coordinated the project. All authors read and approved the final manuscript.

## Supplementary Material

Additional file 1: Table S1 Possible assignment for the IR features observed for fullerols. Click here for file

Additional file 2: Table S2 Correlation *inter se* plant characters and phytomedicine contents, and with plant water content and two-tail *P*-vales (in the second row). Description of data: See above. Click here for file
