# Supplementary figures and images for: Nanobiotechnology can boost crop production and quality: first evidence from increased plant biomass, fruit yield and phytomedicine content in bitter melon (Momordica charantia)
Source: BMC Biotechnol. 2013 Apr 26;13:37. doi: 10.1186/1472-6750-13-37 (PMC3644254; doi:10.1186/1472-6750-13-37)

**Supplementary Table 1** Possible assignment for the IR features observed for fullerols


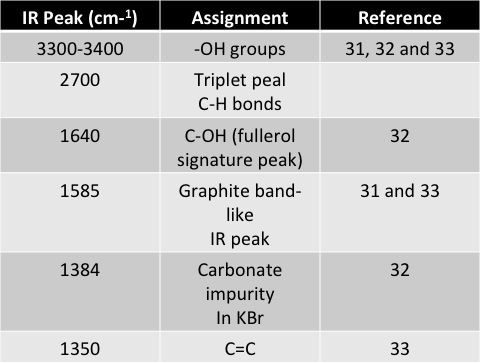

Supplement: Additional file 1: Table S1 — Possible assignment for the IR features observed for fullerols. [file 1472-6750-13-37-S1.doc]
