# Supplementary material for: Nanobiotechnology can boost crop production and quality: first evidence from increased plant biomass, fruit yield and phytomedicine content in bitter melon (Momordica charantia)
Source: BMC Biotechnol. 2013 Apr 26;13:37. doi: 10.1186/1472-6750-13-37 (PMC3644254; doi:10.1186/1472-6750-13-37)
Supplement: Additional file 2: Table S2 — Correlation inter se plant characters and phytomedicine contents, and with plant water content and two-tail P-vales (in the second row). Description of data: See above. [file 1472-6750-13-37-S2.doc]

**Supplementary Table 2** Correlation *inter se* plant characters and phytomedicine contents, and with plant water content and two-tail *P*-vales (in the second row)

| Variables | 2 | 3 | 4 | 5 | 6 | 7 | 8 | 9 | 10 |
| --- | --- | --- | --- | --- | --- | --- | --- | --- | --- |
| 1. Fruit length | 0.962**  0.0021 | 0.195  0.7112 | 0.799  0.0565 | -0.261  0.6174 | 0.179  0.7344 | 0.482  0.3330 | 0.478  0.3376 | -0.295  0.5703 | -0.408  0.4220 |
| 2. Fruit weight |  | 0.368  0.4729 | 0.899*  0.0148 | -0.092  0.8624 | 0.049  0.9266 | 0.366  0.4755 | 0.466  0.3516 | -0.218  0.6782 | -0.195  0.7112 |
| 3. Fruit number |  |  | 0.734  0.0967 | 0.217  0.6796 | -0.273  0.6007 | 0.060  0.9101 | 0.240  0.6469 | 0.152  0.7738 | 0.109  0.8371 |
| 4. Fruit yield |  |  |  | 0.031  0.9535 | -0.100  0.8505 | 0.351  0.4951 | 0.417  0.4108 | -0.096  0.8564 | -0.108  0.8386 |
| 5. Biomass yield |  |  |  |  | -0.033  0.9505 | 0.146  0.7826 | -0.676  0.1405 | 0.784  0.0649 | 0.783  0.0655 |
| 6.Cucurbitacin-B content |  |  |  |  |  | 0.230  0.6611 | 0.205  0.6968 | 0.522  0.2881 | -0.540  0.2687 |
| 7. Lycopene content |  |  |  |  |  |  | -0.370  0.4703 | 0.146  0.7826 | -0.279  0.5924 |
| 8. Charantin content |  |  |  |  |  |  |  | -0.413  0.4157 | -0.583  0.2246 |
| 9. Insulin content |  |  |  |  |  |  |  |  | 0.311  0.5485 |
| 10. Plant water content |  |  |  |  |  |  |  |  |  |

* and ** denote significant at 5% and 1% level, respectively with table values of 0.811 and 0.917, respectively.
